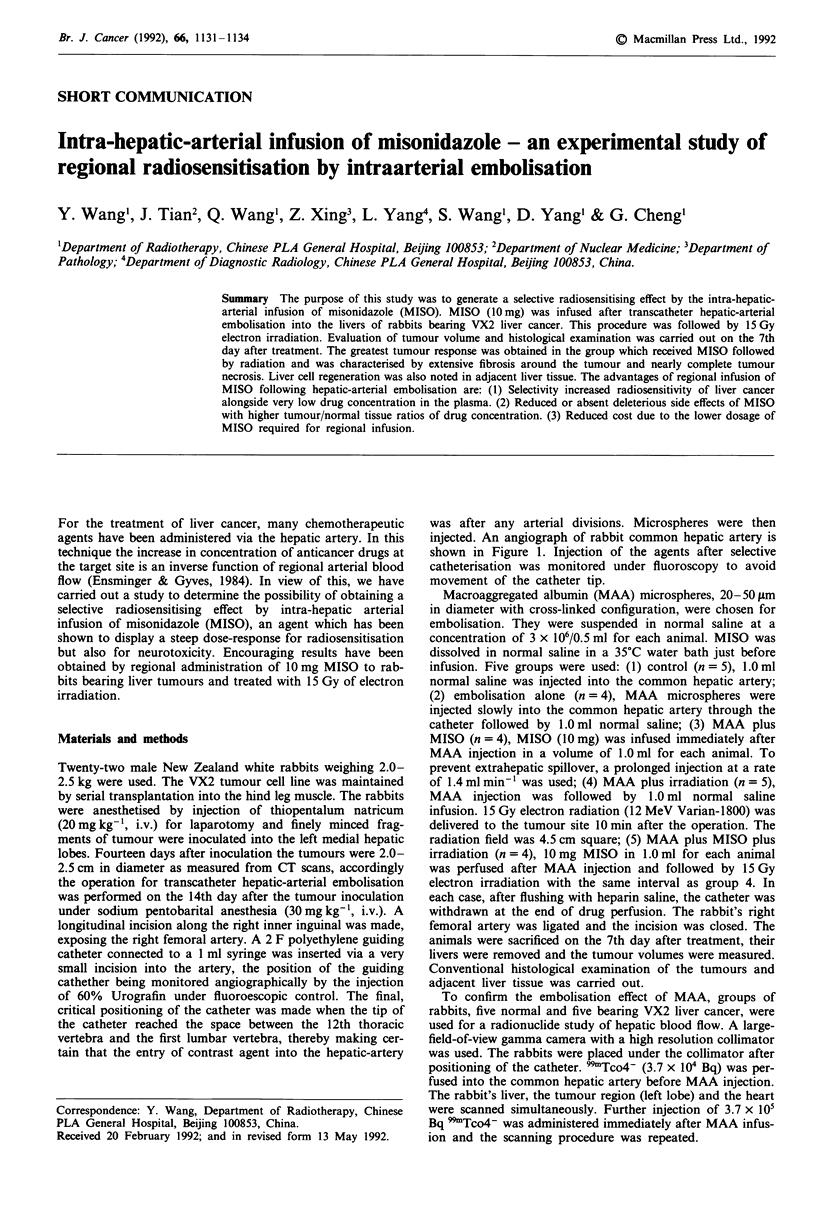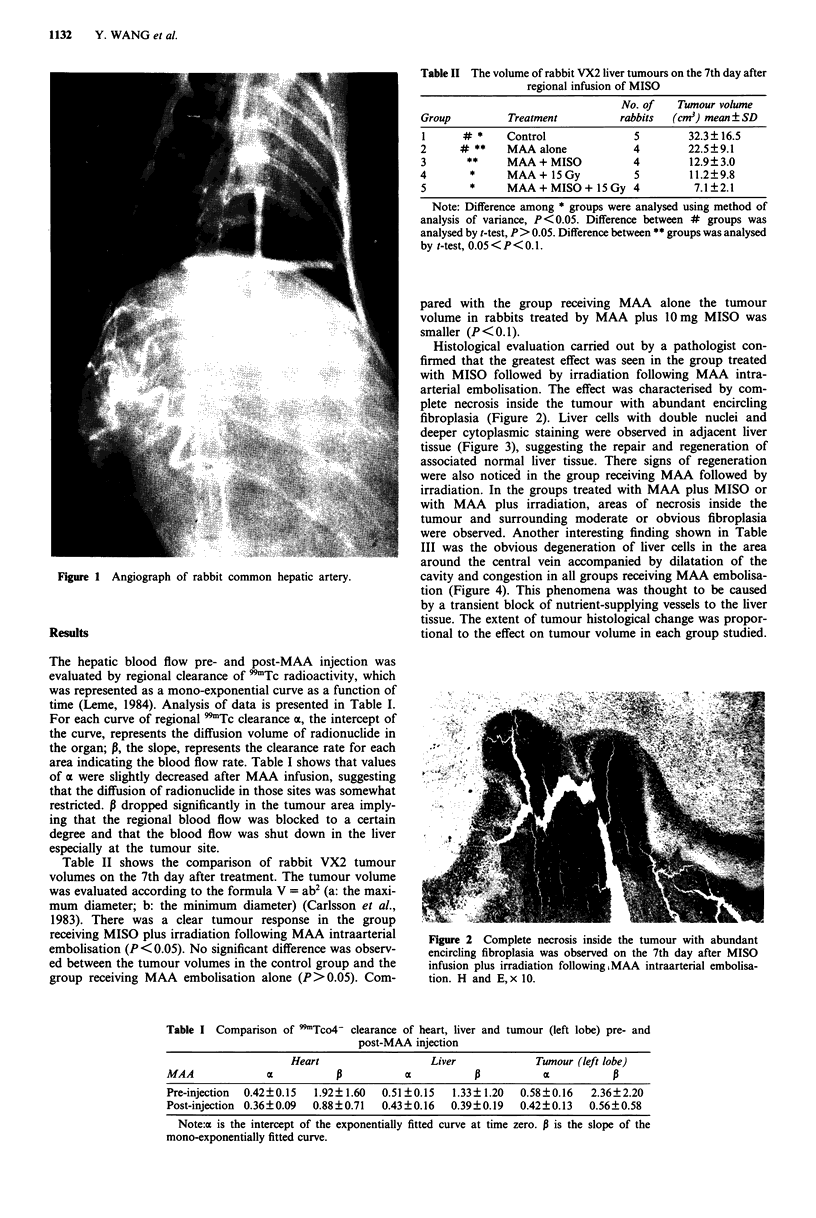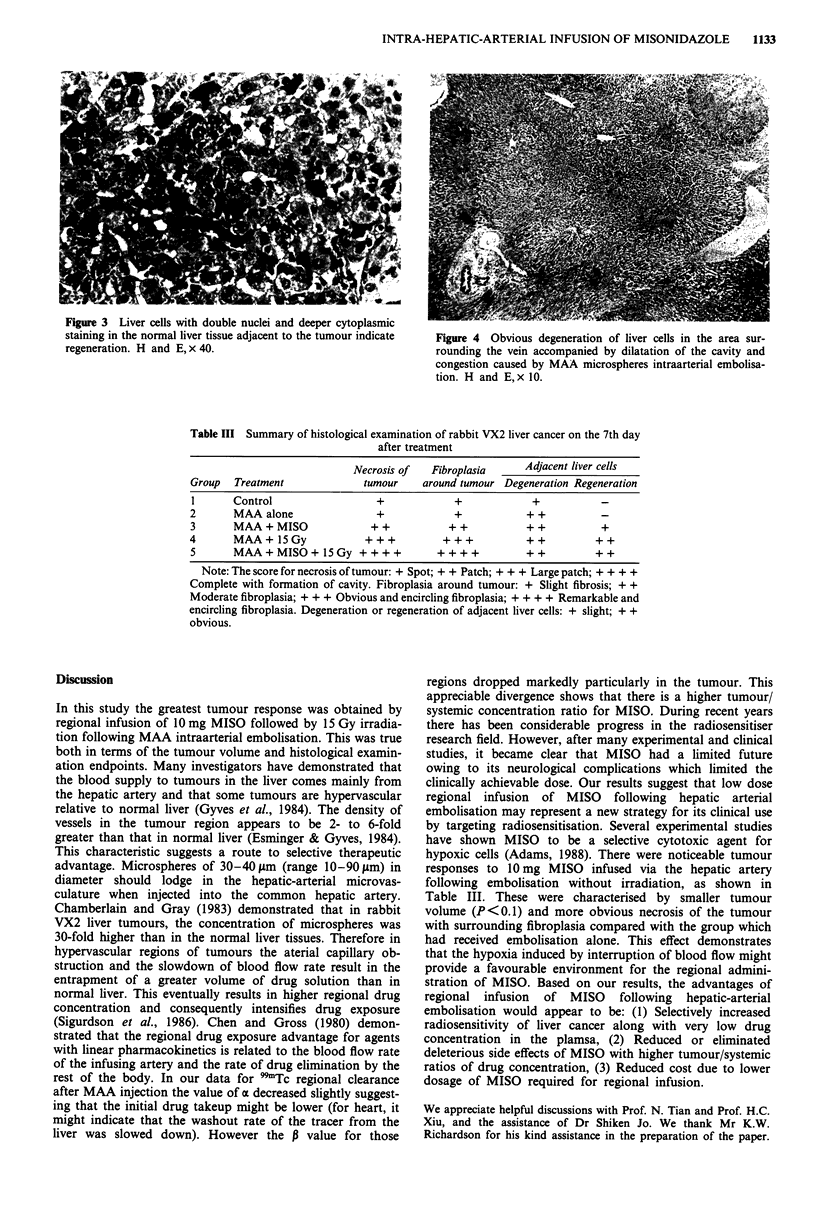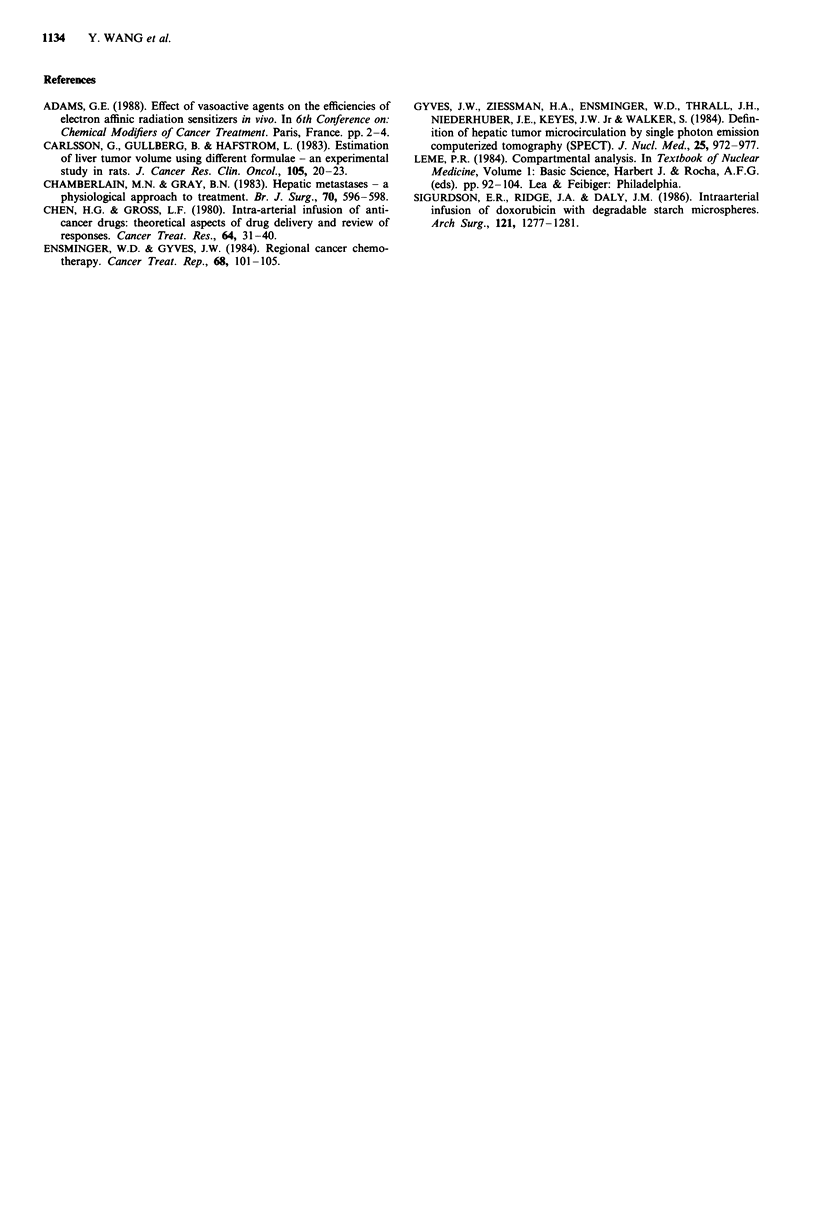# Intra-hepatic-arterial infusion of misonidazole--an experimental study of regional radiosensitisation by intraarterial embolisation.

**DOI:** 10.1038/bjc.1992.422

**Published:** 1992-12

**Authors:** Y. Wang, J. Tian, Q. Wang, Z. Xing, L. Yang, S. Wang, D. Yang, G. Cheng

**Affiliations:** Department of Radiotherapy, Chinese PLA General Hospital, Beijing.

## Abstract

**Images:**


Br. J. Cancer (1992), 66, 1131-1134 © Macmillan Press Ltd., 1992

SHORT COMMUNICATION

### Intra-hepatic-arterial infusion of misonidazole – an experimental study of regional radiosensitisation by intraarterial embolisation

Y. Wang[1], J. Tian[2], Q. Wang[1], Z. Xing[3], L. Yang[4], S. Wang[1], D. Yang[1] & G. Cheng[1]

[1]*Department of Radiotherapy, Chinese PLA General Hospital, Beijing 100853;* [2]*Department of Nuclear Medicine;* [3]*Department of Pathology;* [4]*Department of Diagnostic Radiology, Chinese PLA General Hospital, Beijing 100853, China.*

**Summary** The purpose of this study was to generate a selective radiosensitising effect by the intra-hepatic-arterial infusion of misonidazole (MISO). MISO (10 mg) was infused after transcatheter hepatic-arterial embolisation into the livers of rabbits bearing VX2 liver cancer. This procedure was followed by 15 Gy electron irradiation. Evaluation of tumour volume and histological examination was carried out on the 7th day after treatment. The greatest tumour response was obtained in the group which received MISO followed by radiation and was characterised by extensive fibrosis around the tumour and nearly complete tumour necrosis. Liver cell regeneration was also noted in adjacent liver tissue. The advantages of regional infusion of MISO following hepatic-arterial embolisation are: (1) Selectivity increased radiosensitivity of liver cancer alongside very low drug concentration in the plasma. (2) Reduced or absent deleterious side effects of MISO with higher tumour/normal tissue ratios of drug concentration. (3) Reduced cost due to the lower dosage of MISO required for regional infusion.

For the treatment of liver cancer, many chemotherapeutic agents have been administered via the hepatic artery. In this technique the increase in concentration of anticancer drugs at the target site is an inverse function of regional arterial blood flow (Ensminger & Gyves, 1984). In view of this, we have carried out a study to determine the possibility of obtaining a selective radiosensitising effect by intra-hepatic arterial infusion of misonidazole (MISO), an agent which has been shown to display a steep dose-response for radiosensitisation but also for neurotoxicity. Encouraging results have been obtained by regional administration of 10 mg MISO to rabbits bearing liver tumours and treated with 15 Gy of electron irradiation.

### Materials and methods

Twenty-two male New Zealand white rabbits weighing 2.0–2.5 kg were used. The VX2 tumour cell line was maintained by serial transplantation into the hind leg muscle. The rabbits were anesthetised by injection of thiopentalum natricum (20 mg $kg^{-1}$, i.v.) for laparotomy and finely minced fragments of tumour were inoculated into the left medial hepatic lobes. Fourteen days after inoculation the tumours were 2.0–2.5 cm in diameter as measured from CT scans, accordingly the operation for transcatheter hepatic-arterial embolisation was performed on the 14th day after the tumour inoculation under sodium pentobarital anesthesia (30 mg $kg^{-1}$, i.v.). A longitudinal incision along the right inner inguinal was made, exposing the right femoral artery. A 2 F polyethylene guiding catheter connected to a 1 ml syringe was inserted via a very small incision into the artery, the position of the guiding cathether being monitored angiographically by the injection of 60% Urografin under fluoroescopic control. The final, critical positioning of the catheter was made when the tip of the catheter reached the space between the 12th thoracic vertebra and the first lumbar vertebra, thereby making certain that the entry of contrast agent into the hepatic-artery was after any arterial divisions. Microspheres were then injected. An angiograph of rabbit common hepatic artery is shown in Figure 1. Injection of the agents after selective catheterisation was monitored under fluoroscopy to avoid movement of the catheter tip.

Macroaggregated albumin (MAA) microspheres, 20–50 μm in diameter with cross-linked configuration, were chosen for embolisation. They were suspended in normal saline at a concentration of $3 \times 10^6$/0.5 ml for each animal. MISO was dissolved in normal saline in a 35°C water bath just before infusion. Five groups were used: (1) control ($n = 5$), 1.0 ml normal saline was injected into the common hepatic artery; (2) embolisation alone ($n = 4$), MAA microspheres were injected slowly into the common hepatic artery through the catheter followed by 1.0 ml normal saline; (3) MAA plus MISO ($n = 4$), MISO (10 mg) was infused immediately after MAA injection in a volume of 1.0 ml for each animal. To prevent extrahepatic spillover, a prolonged injection at a rate of 1.4 ml $min^{-1}$ was used; (4) MAA plus irradiation ($n = 5$), MAA injection was followed by 1.0 ml normal saline infusion. 15 Gy electron radiation (12 MeV Varian-1800) was delivered to the tumour site 10 min after the operation. The radiation field was 4.5 cm square; (5) MAA plus MISO plus irradiation ($n = 4$), 10 mg MISO in 1.0 ml for each animal was perfused after MAA injection and followed by 15 Gy electron irradiation with the same interval as group 4. In each case, after flushing with heparin saline, the catheter was withdrawn at the end of drug perfusion. The rabbit's right femoral artery was ligated and the incision was closed. The animals were sacrificed on the 7th day after treatment, their livers were removed and the tumour volumes were measured. Conventional histological examination of the tumours and adjacent liver tissue was carried out.

To confirm the embolisation effect of MAA, groups of rabbits, five normal and five bearing VX2 liver cancer, were used for a radionuclide study of hepatic blood flow. A large-field-of-view gamma camera with a high resolution collimator was used. The rabbits were placed under the collimator after positioning of the catheter. $^{99m}Tco4^-$ ($3.7 \times 10^4$ Bq) was perfused into the common hepatic artery before MAA injection. The rabbit's liver, the tumour region (left lobe) and the heart were scanned simultaneously. Further injection of $3.7 \times 10^5$ Bq $^{99m}Tco4^-$ was administered immediately after MAA infusion and the scanning procedure was repeated.

Correspondence: Y. Wang, Department of Radiotherapy, Chinese PLA General Hospital, Beijing 100853, China.
Received 20 February 1992; and in revised form 13 May 1992.

**Figure 1** Angiograph of rabbit common hepatic artery.

### Results

The hepatic blood flow pre- and post-MAA injection was evaluated by regional clearance of $^{99m}$Tc radioactivity, which was represented as a mono-exponential curve as a function of time (Leme, 1984). Analysis of data is presented in Table I. For each curve of regional $^{99m}$Tc clearance $\alpha$, the intercept of the curve, represents the diffusion volume of radionuclide in the organ; $\beta$, the slope, represents the clearance rate for each area indicating the blood flow rate. Table I shows that values of $\alpha$ were slightly decreased after MAA infusion, suggesting that the diffusion of radionuclide in those sites was somewhat restricted. $\beta$ dropped significantly in the tumour area implying that the regional blood flow was blocked to a certain degree and that the blood flow was shut down in the liver especially at the tumour site.

Table II shows the comparison of rabbit VX2 tumour volumes on the 7th day after treatment. The tumour volume was evaluated according to the formula $V = ab^2$ (a: the maximum diameter; b: the minimum diameter) (Carlsson *et al.*, 1983). There was a clear tumour response in the group receiving MISO plus irradiation following MAA intraarterial embolisation ($P<0.05$). No significant difference was observed between the tumour volumes in the control group and the group receiving MAA embolisation alone ($P>0.05$). Compared with the group receiving MAA alone the tumour volume in rabbits treated by MAA plus 10 mg MISO was smaller ($P<0.1$).

**Table II** The volume of rabbit VX2 liver tumours on the 7th day after regional infusion of MISO

| *Group* | | *Treatment* | *No. of rabbits* | *Tumour volume (cm³) mean ± SD* |
|---|---|---|---|---|
| 1 | # * | Control | 5 | 32.3±16.5 |
| 2 | # ** | MAA alone | 4 | 22.5±9.1 |
| 3 | ** | MAA + MISO | 4 | 12.9±3.0 |
| 4 | * | MAA + 15 Gy | 5 | 11.2±9.8 |
| 5 | * | MAA + MISO + 15 Gy | 4 | 7.1±2.1 |

Note: Difference among * groups were analysed using method of analysis of variance, $P<0.05$. Difference between # groups was analysed by *t*-test, $P>0.05$. Difference between ** groups was analysed by *t*-test, $0.05<P<0.1$.

Histological evaluation carried out by a pathologist confirmed that the greatest effect was seen in the group treated with MISO followed by irradiation following MAA intraarterial embolisation. The effect was characterised by complete necrosis inside the tumour with abundant encircling fibroplasia (Figure 2). Liver cells with double nuclei and deeper cytoplasmic staining were observed in adjacent liver tissue (Figure 3), suggesting the repair and regeneration of associated normal liver tissue. There signs of regeneration were also noticed in the group receiving MAA followed by irradiation. In the groups treated with MAA plus MISO or with MAA plus irradiation, areas of necrosis inside the tumour and surrounding moderate or obvious fibroplasia were observed. Another interesting finding shown in Table III was the obvious degeneration of liver cells in the area around the central vein accompanied by dilatation of the cavity and congestion in all groups receiving MAA embolisation (Figure 4). This phenomena was thought to be caused by a transient block of nutrient-supplying vessels to the liver tissue. The extent of tumour histological change was proportional to the effect on tumour volume in each group studied.

**Figure 2** Complete necrosis inside the tumour with abundant encircling fibroplasia was observed on the 7th day after MISO infusion plus irradiation following MAA intraarterial embolisation. H and E, × 10.

**Table I** Comparison of $^{99m}Tco4^-$ clearance of heart, liver and tumour (left lobe) pre- and post-MAA injection

| | *Heart* | | *Liver* | | *Tumour (left lobe)* | |
|---|---|---|---|---|---|---|
| *MAA* | $\alpha$ | $\beta$ | $\alpha$ | $\beta$ | $\alpha$ | $\beta$ |
| Pre-injection | 0.42±0.15 | 1.92±1.60 | 0.51±0.15 | 1.33±1.20 | 0.58±0.16 | 2.36±2.20 |
| Post-injection | 0.36±0.09 | 0.88±0.71 | 0.43±0.16 | 0.39±0.19 | 0.42±0.13 | 0.56±0.58 |

Note: $\alpha$ is the intercept of the exponentially fitted curve at time zero. $\beta$ is the slope of the mono-exponentially fitted curve.

**Figure 3** Liver cells with double nuclei and deeper cytoplasmic staining in the normal liver tissue adjacent to the tumour indicate regeneration. H and E, × 40.

**Figure 4** Obvious degeneration of liver cells in the area surrounding the vein accompanied by dilatation of the cavity and congestion caused by MAA microspheres intraarterial embolisation. H and E, × 10.

**Table III** Summary of histological examination of rabbit VX2 liver cancer on the 7th day after treatment

| *Group* | *Treatment* | *Necrosis of tumour* | *Fibroplasia around tumour* | *Adjacent liver cells* | |
|---|---|---|---|---|---|
| | | | | *Degeneration* | *Regeneration* |
| 1 | Control | + | + | + | – |
| 2 | MAA alone | + | + | + + | – |
| 3 | MAA + MISO | + + | + + | + + | + |
| 4 | MAA + 15 Gy | + + + | + + + | + + | + + |
| 5 | MAA + MISO + 15 Gy | + + + + | + + + + | + + | + + |

Note: The score for necrosis of tumour: + Spot; + + Patch; + + + Large patch; + + + + Complete with formation of cavity. Fibroplasia around tumour: + Slight fibrosis; + + Moderate fibroplasia; + + + Obvious and encircling fibroplasia; + + + + Remarkable and encircling fibroplasia. Degeneration or regeneration of adjacent liver cells: + slight; + + obvious.

### Discussion

In this study the greatest tumour response was obtained by regional infusion of 10 mg MISO followed by 15 Gy irradiation following MAA intraarterial embolisation. This was true both in terms of the tumour volume and histological examination endpoints. Many investigators have demonstrated that the blood supply to tumours in the liver comes mainly from the hepatic artery and that some tumours are hypervascular relative to normal liver (Gyves *et al.*, 1984). The density of vessels in the tumour region appears to be 2- to 6-fold greater than that in normal liver (Esminger & Gyves, 1984). This characteristic suggests a route to selective therapeutic advantage. Microspheres of 30–40 μm (range 10–90 μm) in diameter should lodge in the hepatic-arterial microvasculature when injected into the common hepatic artery. Chamberlain and Gray (1983) demonstrated that in rabbit VX2 liver tumours, the concentration of microspheres was 30-fold higher than in the normal liver tissues. Therefore in hypervascular regions of tumours the aterial capillary obstruction and the slowdown of blood flow rate result in the entrapment of a greater volume of drug solution than in normal liver. This eventually results in higher regional drug concentration and consequently intensifies drug exposure (Sigurdson *et al.*, 1986). Chen and Gross (1980) demonstrated that the regional drug exposure advantage for agents with linear pharmacokinetics is related to the blood flow rate of the infusing artery and the rate of drug elimination by the rest of the body. In our data for $^{99m}$Tc regional clearance after MAA injection the value of α decreased slightly suggesting that the initial drug takeup might be lower (for heart, it might indicate that the washout rate of the tracer from the liver was slowed down). However the β value for those regions dropped markedly particularly in the tumour. This appreciable divergence shows that there is a higher tumour/systemic concentration ratio for MISO. During recent years there has been considerable progress in the radiosensitiser research field. However, after many experimental and clinical studies, it became clear that MISO had a limited future owing to its neurological complications which limited the clinically achievable dose. Our results suggest that low dose regional infusion of MISO following hepatic arterial embolisation may represent a new strategy for its clinical use by targeting radiosensitisation. Several experimental studies have shown MISO to be a selective cytotoxic agent for hypoxic cells (Adams, 1988). There were noticeable tumour responses to 10 mg MISO infused via the hepatic artery following embolisation without irradiation, as shown in Table III. These were characterised by smaller tumour volume ($P<0.1$) and more obvious necrosis of the tumour with surrounding fibroplasia compared with the group which had received embolisation alone. This effect demonstrates that the hypoxia induced by interruption of blood flow might provide a favourable environment for the regional administration of MISO. Based on our results, the advantages of regional infusion of MISO following hepatic-arterial embolisation would appear to be: (1) Selectively increased radiosensitivity of liver cancer along with very low drug concentration in the plamsa, (2) Reduced or eliminated deleterious side effects of MISO with higher tumour/systemic ratios of drug concentration, (3) Reduced cost due to lower dosage of MISO required for regional infusion.

We appreciate helpful discussions with Prof. N. Tian and Prof. H.C. Xiu, and the assistance of Dr Shiken Jo. We thank Mr K.W. Richardson for his kind assistance in the preparation of the paper.

### References

ADAMS, G.E. (1988). Effect of vasoactive agents on the efficiencies of electron affinic radiation sensitizers *in vivo*. In *6th Conference on: Chemical Modifiers of Cancer Treatment*. Paris, France. pp. 2–4.

CARLSSON, G., GULLBERG, B. & HAFSTROM, L. (1983). Estimation of liver tumor volume using different formulae – an experimental study in rats. *J. Cancer Res. Clin. Oncol.*, **105**, 20–23.

CHAMBERLAIN, M.N. & GRAY, B.N. (1983). Hepatic metastases – a physiological approach to treatment. *Br. J. Surg.*, **70**, 596–598.

CHEN, H.G. & GROSS, L.F. (1980). Intra-arterial infusion of anti-cancer drugs: theoretical aspects of drug delivery and review of responses. *Cancer Treat. Res.*, **64**, 31–40.

ENSMINGER, W.D. & GYVES, J.W. (1984). Regional cancer chemotherapy. *Cancer Treat. Rep.*, **68**, 101–105.

GYVES, J.W., ZIESSMAN, H.A., ENSMINGER, W.D., THRALL, J.H., NIEDERHUBER, J.E., KEYES, J.W. Jr & WALKER, S. (1984). Definition of hepatic tumor microcirculation by single photon emission computerized tomography (SPECT). *J. Nucl. Med.*, **25**, 972–977.

LEME, P.R. (1984). Compartmental analysis. In *Textbook of Nuclear Medicine*, Volume 1: Basic Science, Harbert J. & Rocha, A.F.G. (eds). pp. 92–104. Lea & Feibiger: Philadelphia.

SIGURDSON, E.R., RIDGE, J.A. & DALY, J.M. (1986). Intraarterial infusion of doxorubicin with degradable starch microspheres. *Arch Surg.*, **121**, 1277–1281.